# Substance use and mental health factors associated with self-reported higher risk cannabis use among people with HIV screened in primary care

**DOI:** 10.1186/s12889-025-23735-8

**Published:** 2025-07-29

**Authors:** Maha N. Mian, V. Sarovar, T. Levine, A. Lea, A. Leibowitz, M. Luu, J. Flamm, C. B. Hare, M. Horberg, K. C. Young-Wolff, K. T. Phillips, M. J. Silverberg, D. D. Satre

**Affiliations:** 1 Department of Psychology, Suffolk University, 73 Tremont St, #8031, Boston, MA 02108 USA; 2 Division of Research, Kaiser Permanente Northern California, Pleasanton, California, CA USA; 3 Department of Psychiatry and Behavioral Sciences, Weill Institute for Neurosciences, University of California, San Francisco, CA USA; 4 Kaiser Permanente Oakland Medical Center, Oakland, CA USA; 5 Kaiser Permanente Sacramento Medical Center, Sacramento, CA USA; 6 Kaiser Permanente San Francisco Medical Center, San Francisco, CA USA; 7 Mid-Atlantic Permanente Research Institute, Kaiser Permanente Mid-Atlantic States, Rockville, MD USA; 8 Center for Integrated Health Care Research, Kaiser Permanente Hawaii, Honolulu, HI USA; 9 Department of Health Systems Science, Kaiser Permanente Bernard J. Tyson School of Medicine, Pasadena, CA USA

**Keywords:** Cannabis, Marijuana, HIV, Primary care

## Abstract

**Background:**

While cannabis use is prevalent among people with HIV (PWH), factors associated with higher-risk use require further study. We examined factors (mental health, sociodemographics, substance use, HIV clinical markers) associated with risk for cannabis use disorder (CUD) among PWH who used cannabis.

**Methods:**

Participants included adult (≥ 18 years old) PWH from 3 HIV primary care clinics in Kaiser Permanente Northern California who reported past three-month cannabis use through the computerized Tobacco, Alcohol, Prescription medication, and other Substance use (TAPS) screening. Primary outcome was TAPS cannabis score (range 1–3), categorized as any use (1) and higher risk for CUD (≥ 2). Measures included sociodemographics (age, sex, race, neighborhood deprivation index [NDI]), duration of HIV status, Charlson Comorbidity Index (CCI), HIV RNA, CD4 cell counts, higher risk tobacco use (TAPS tobacco score ≥ 2), depression, and anxiety symptoms. Unadjusted and multivariable logistic regression examined factors associated with higher risk for CUD.

**Results:**

Of the complete sample (*N* = 973; 94.1% Male; 58.5% White; Age _Median_=54.5), 35.9% reported higher risk for CUD. Unadjusted models indicated age, Black race, higher CCI, depression, anxiety, and higher risk tobacco use were associated with higher risk, while only Black race (OR = 1.90, 95% CI[1.32, 2.72]), anxiety (OR = 1.91, 95% CI[1.22, 2.99]), and higher risk tobacco use (OR = 2.25, 95% CI[1.46, 3.48]) remained significant in the multivariable model.

**Conclusions:**

Black race, anxiety and tobacco use were associated with higher risk for CUD among PWH in a multivariable model. Clinical efforts to screen and provide interventions for preventing CUD alongside anxiety and tobacco use among PWH should be evaluated.

## Introduction

Cannabis use is prevalent in the United States, particularly among people living with HIV (PWH) Compared with 45% of adults without HIV, a nationally representative study found that 77% of PWH reported lifetime cannabis use [1]. Another study of over 10,000 PWH drawn from the Center for AIDS Research Network of Integrated Clinical Systems found that 31% met criteria for Cannabis Use Disorder (CUD), defined as problematic cannabis use resulting in functional impairment [2].

These high rates of cannabis use are concerning given negative consequences associated with use [3, 4]. While factors typically associated with any use are known, further research is required to determine which factors predict higher risk cannabis use among PWH. For example, PWH might have higher risk for CUD given the increased prevalence of mental health concerns in this population, although associations between cannabis use and depression and anxiety symptoms among PWH are not consistent [5]. HIV viral control (e.g., HIV RNA) may be another important factor related to cannabis use, but associations between HIV markers (i.e., HIV RNA and CD4) and cannabis use are also mixed [6]. One study found among PWH using cannabis, use was not associated with durable viral suppression [7], while another found that cannabis use was associated with having detectable HIV RNA, though CD4 levels were comparable [6]. Long-term heavy cannabis use was also found to be unrelated to HIV RNA, CD4, cancer, or mortality among men with HIV [8]. Furthermore, PWH may have therapeutic reasons for using cannabis to address HIV-related symptoms [9–12]. Presently, the assessment of cannabis use severity in conjunction with medical and psychiatric factors is often lacking in existing studies. Standardized clinical assessments of cannabis use severity within a healthcare setting could provide important insights about the links between HIV markers, medical, and psychiatric factors, and cannabis use among PWH.

Identification of PWH at elevated risk for CUD requires further elucidation. The present study aimed to examine predictors of higher risk for CUD among PWH who reported recent cannabis use on a digital screening tool in HIV primary care settings in an integrated healthcare system. First, we developed a descriptive profile of PWH who reported any cannabis use in the prior three months. Second, we examined whether socio-demographic factors, other substance use (tobacco, alcohol), depression, anxiety, and HIV RNA were associated with being at higher risk for CUD at the time of screening. We hypothesized that, descriptively, PWH with mental health conditions, more elevated HIV biomarkers, and those reporting higher risk for other substance use would be more represented at higher frequencies in the higher risk for CUD group. Similarly, we hypothesized that these factors would be associated with greater likelihood of higher risk for CUD.

## Methods

### Study design and sample

This study was based in Kaiser Permanente Northern California (KPNC), an integrated healthcare delivery system providing primary and specialty care to over 4.5 million people, including approximately 5,000 PWH across the three largest KPNC HIV primary care clinics in Oakland, San Francisco, and Sacramento. The Promoting Access to Care Engagement (PACE) trial examined mental health, substance use, and treatment outcomes for PWH following electronic screening combined with behavioral interventions in the three clinics. The study protocol was published previously [13]. All procedures were in accordance with and approved by the KPNC and University of California, San Francisco Institutional Review Boards, including waiver of informed consent to examine participant medical records.

The study sample included adult PWH who reported cannabis use in the prior three months based on screening delivered through the KPNC patient portal (KP.org) in the two weeks prior to an outpatient primary care appointment or during that appointment via clinic tablets between October 30, 2018 and July 17, 2020. Questionnaire results were automatically uploaded into the electronic health record (EHR) and available to clinicians during visits.

### Measures

The primary study outcome was screening for higher risk for CUD, identified with the publicly available Tobacco, Alcohol, Prescription medication, and other Substance use (TAPS) Tool, a computerized, self-administered instrument [14]. The TAPS was developed as a screening tool through the National Drug Abuse Treatment Clinical Trials Network and validated for use in primary care settings among diverse samples, demonstrating good sensitivity and specificity for assessing problem alcohol, tobacco, and cannabis use [15, 16]. For identifying cannabis use problems, sensitivity has been estimated at 0.82 and specificity at 0.93. Prior work published from our parent study has examined the implementation of the TAPS as a screening tool in primary care settings specifically for PWH [13, 17, 18]. TAPS substance use scores range from 0 to 4 for alcohol and 0–3 for tobacco and cannabis. Participants who reported any tobacco, alcohol, or cannabis use in the past three months received a respective TAPS score of 1, and a TAPS score of ≥ 2 was considered higher risk for a substance use disorder (SUD) for that substance, per TAPS scoring guidelines. Participants who used cannabis in the past three months answered follow-up items: “In the past 3 months, have you had a strong desire or urge to use marijuana at least once a week or more often?” and “In the past 3 months, has anyone expressed concern about your use of marijuana?”.

Demographic variables were collected through the EHR, including age at screening, sex, race/ethnicity, and neighborhood deprivation index (NDI), which is a census-informed indicator of socioeconomic status [19]. Comorbidities were assessed by the Charlson Comorbidity Index (CCI), extracted from EHR and modified to remove HIV/AIDS from the index [20]. Duration of time from HIV diagnosis to screening, and HIV RNA (log_10_ transformed) and CD4 + T cell count (CD4; cells/µL) within six months of screening were also collected from the EHR and the KPNC HIV Registry, following National HIV treatment guidelines for use [13, 21].

SUD, depression, and anxiety disorder diagnoses were based on ICD-10 criteria during the period 12 months prior to screening and were extracted from EHR. SUD included any CUD diagnosis prior to screening. Additionally, CUD diagnosis either 12 months prior to or 12 months after the screening visit was also included. Depression and anxiety symptoms were assessed with the Patient Health Questionnaire (PHQ-9) and General Anxiety Disorder (GAD-2), respectively [22, 23] concurrent with TAPS administration. Both the PHQ-9 and GAD-2 are well-established mental health screening measures for both HIV samples and primary care settings [24–26]. Positive depression score was defined as a PHQ-9 score ≥ 10 and anxiety as a GAD-2 score ≥ 3, in accordance with previously validated cutoff criteria [23, 27].

### Data analytic plan

First, we performed descriptive analyses and compared PWH with self-reported higher risk for CUD (Cannabis TAPS score = 2+) and those reporting any past three-month use (Cannabis TAPS = 1). Group differences were assessed with t-tests and chi-square tests. Second, we conducted unadjusted logistic regression models for each predictor and the outcome of scoring at higher risk for CUD. Finally, significant unadjusted predictors were included in a multivariable regression model. Reference group for age (31–40) was selected to examine cannabis use among younger and older adults, given associations between cannabis use and negative consequences among younger adults and the increased rates of cannabis use initiation among older adults [28, 29]. Analyses were conducted on SPSS v. 29.

## Results

### Study sample

Adult PWH completed 3,903 questionnaires during the study period. Screenings were excluded for: being associated with cancellations or no-show visits (*n* = 6); having no associated eligible visit (*n* = 68); and repeated screenings (*n* = 1000). Finally, PWH not reporting recent cannabis use at screening (*n* = 1856) were removed, leaving 973 unique PWH.

The majority of participants were male (sex at birth; 94.1%) and the median age of participants was 54.5 years old (Table 1). More than half of participants were White (58.5%). Participants were distributed across levels of neighborhood deprivation, with slightly more participants in the least deprived category. Average log10 HIV RNA was 1.8 (*SD* = 0.5).Average CD4 cells per *u*L was 666.9 (*SD* = 290.4). On the depression screen (PHQ-9), 15.7% of participants scored positive (10+), while on the anxiety screen (GAD-2), 16% scored positive (3+). PWH had prior depression (20%), anxiety (18.3%), and SUD diagnoses (19.1%), and 27.6% and 10.4% of participants reported higher risk alcohol and tobacco use, respectively. Among the whole sample, 92 PWH were diagnosed with a CUD (4.5%).

**Table 1:** Characteristics of people with HIV reporting past 3-month cannabis use through screening in primary care

	Complete sample	Sample by TAPS Cannabis Score		
	*N* = 973	Higher Risk for CUD [Cannabis TAPS ≥ 2] (*n* = 349)	Any Use [Cannabis TAPS = 1] (*n* = 624)	Group Differences (t, F)
Age in years, N (%)				***p*** **=.003**
17–30	76 (7.8)	37 (10.6)	39 (6.3)	
31–40	137 (14.1)	49 (14.0)	88 (14.1)	
41–50	153 (15.7)	60 (17.2)	93 (14.9)	
51–60	306 (31.4)	84 (24.1)	222 (35.6)	
61–70	231 (23.7)	89 (25.5)	142 (22.8)	
71+	70 (7.2)	30 (8.6)	40 (6.4)	
Sex, N (%)				
Male	916 (94.1)	326 (93.4)	590 (94.6)	*p* >.05
Female	57 (5.9)	23 (6.6)	34 (5.4)	
Race/Ethnicity, N (%)				***p*** **<.001**
Asian	49 (5.0)	12 (3.4)	37 (5.9)	
Non-Hispanic Black	190 (19.5)	96 (27.5)	94 (15.1)	
Hispanic	131 (13.5)	42 (12.0)	89 (14.3)	
Non-Hispanic White	569 (58.5)	188 (53.9)	381 (61.1)	
Other/UK	34 (3.5)	11 (3.2)	23 (3.7)	
NDI quartile, N (%)				***p*** **=.018**
1 (least deprived)	309 (31.8)	98 (28.1)	211 (33.8)	
2	227 (23.3)	75 (21.5)	152 (24.4)	
3	216 (22.2)	78 (22.3)	138 (22.1)	
4 (most deprived)	221(22.7)	98 (28.1)	123 (19.7)	
CUD Diagnosis (12 months pre/post screening)	92 (4.5)	38 (10.9)	28 (4.5)	*p* <.001
HIV duration (years between diagnosis and screening), mean (SD)	17.9 (10.4)	17.7 (10.8)	18.1 (10.2)	*p* >.05
Charlson Comorbidity Index, mean (SD)	0.82 (1.5)	0.97 (1.7)	0.74 (1.4)	***p*** **=.016**
PHQ-9 (≥ 10), N (%)	153 (15.7)	67 (19.2)	86 (13.8)	***p*** **=.026**
GAD-2 (≥ 3), N (%)	156 (16.0)	77 (22.1)	79 (12.7)	***p*** **<.001**
Higher Risk Tobacco Use, N (%)	101 (10.4)	56 (160)	45 (7.2)	***p*** **<.001**
Higher Risk Alcohol Use, N (%)	269 (27.6)	99 (28.4)	170 (27.2)	*p* >.05
Prior Depression Diagnosis, N (%)	195 (20.0)	81 (23.2)	114 (18.3)	*p* >.05
Prior Anxiety Diagnosis, N (%)	178 (18.3)	75 (21.5)	103 (16.5)	*p* >.05
Prior SUD Diagnosis, N (%)	186 (19.1)	87 (24.9)	99 (15.9)	*p* >.05
HIV RNA Log10, mean (SD)	1.8 (0.5)	1.8 (0.6)	1.8 (0.5)	*p* >.05
CD4 + T cells/*uL*, mean (SD)	666.9 (290.3)	675.8 (326.3)	661.9(267.8)	*p* >.05

Tobacco, Alcohol, Prescription medication, and other Substance use (TAPS) Tool administered through an electronic screen prior to or during clinic visits. Primary outcome was cannabis score (range 0-3), categorized as non- higher risk use (1) and higher risk for cannabis use disorder (Higher risk for CUD) (≥2). NDI = Neighborhood Deprivation Index; CUD= Cannabis Use Disorder, with any CUD diagnosis given 12 months pre or post TAPS screening visit; PHQ-9 = Patient Health Questionnaire, positive screen with a score of 10 or greater; GAD-2 = General Anxiety Disorder, positive screen with score of 3 or greater; HIV RNA log 10 transformed; SUD= Substance Use Disorder; Higher Risk Tobacco Use and Higher Risk Alcohol Use defined as Tobacco or Alcohol TAPS Score =≥2. Group differences between TAPS 1 vs TAPS 2+ assessed with t-tests and chi-square; test-statistics and *p* values reported for significant differences

Item-level frequences on the cannabis TAPS items indicated that 345 respondents (43.5%) endorsed strong desire/urge to use and 45 (5.7%) endorsed having someone express concern about their use. Compared with those reporting any past three-month use, those at higher risk for CUD were also majority male and White (Table 1), but had a greater frequency of Black PWH and a lower frequency of individuals in the least deprived category of the NDI. Those at higher risk for CUD were also more likely to screen positive for depression, anxiety, and tobacco use, and have prior diagnoses for depression, anxiety, and SUD, including a CUD diagnosis.

### Regressions for higher risk for CUD

In unadjusted models (Table 2), significant factors associated with higher CUD risk included: age; Black race (compared with White race; OR = 2.07; 95%CI = 1.48, 2.89); higher CCI score (OR = 1.10; 95%CI = 1.01, 1.20) per 1 point higher; anxiety (OR = 1.95; 95%CI = 1.38, 2.76); depression (OR = 1.49; 95% CI = 1.05, 2.11); and higher risk tobacco use (OR = 2.46; 95%CI = 1.62, 3.73). In the multivariable model, PWH who were Black (compared with White; OR = 1.90, 95%CI = 1.32, 2.72), PWH with anxiety (OR = 1.91, 95%CI = 1.22, 2.99) and higher risk tobacco use (OR = 2.25; 95%CI = 1.46, 3.48) had greater odds for higher risk for CUD. Depression, CCI and age were not associated with reporting higher risk for CUD in the multivariable model.

**Table 2:** Demographic and clinical predictors of scoring at higher risk for cannabis use disorder among people with HIV

	Unadjusted		Multivariable	
Predictors	OR	95% CI	OR	95% CI
Age				
17-30	1.70	(0.96, 3.01)	1.66	(0.92, 3.01)
31-40 (Reference)				
41-50	1.16	(0.72, 1.87)	1.23	(0.74, 2.03)
51-60	0.68	(0.44, 1.05)	0.75	(0.47, 1.20)
61-70	1.13	(0.73, 1.75)	1.33	(0.81, 2.20)
71+	1.35	(0.75, 2.43)	1.61	(0.83, 3.10)
Race/ethnicity				
White (Reference)				
Hispanic	0.96	(0.64, 1.44)	1.03	(0.66, 1.61)
Black	**2.07**	**(1.48, 2.89)**	**1.90**	**1.32, 2.72)**
Asian	0.66	(0.34, 1.29)	0.65	(0.32, 1.32)
Other/Unknown	0.97	(0.46, 2.03)	0.85	(0.38, 1.88)
Sex (Male)	0.82	(0.47, 1.41)		
HIV duration	1.00	(0.99, 1.01)		
Charlson Comorbidity Index	**1.10**	**(1.01, 1.20)**	1.07	(0.97, 1.18)
GAD-2 (3+)	**1.95**	**(1.38, 2.76)**	**1.91**	**(1.22, 2.99)**
PHQ-9 (10+)	**1.49**	**(1.05, 2.11)**	0.93	(0.59, 1.46)
Higher Risk Tobacco Use	**2.46**	**(1.62, 3.73)**	**2.25**	**(1.46, 3.48)**
Higher Risk Alcohol Use	1.06	(0.79, 1.42)	-	-
Prior Depression Diagnosis	1.20	(0.70, 2.06)	-	**-**
Prior Anxiety Diagnosis	1.46	(0.85, 2.51)	-	**-**
Prior SUD Diagnosis	1.50	(0.80, 2.79)	-	**-**
CD4+ T cells/* uL* (per 100 cells)	1.00	(1.00, 1.001)	-	**-**
HIV RNA (per 1 log)	1.20	(0.94, 1.53)	-	**-**

Unadjusted regression models predicting higher risk cannabis use (Cannabis TAPS≥2); significant variables from significant unadjusted models entered in multivariable regression model (age, race, GAD, higher risk tobacco use, Charlson Comorbidity Index, PHQ-9)

Bolded values indicate significance at *p *<.05; *OR* Odds ratio, *CI* Confidence Interval, *GAD* General Anxiety Disorder, *PHQ-9* Patient Health Questionnaire, *SUD* Substance Use Disorder, HIV RNA, log 10 transformed; Higher Risk Tobacco Use and Higher Risk Alcohol Use defined as Tobacco and Alcohol TAPS Score≥2; PHQ-9 defined as ≥10; GAD-2 score defined as ≥3

## Discussion

The present study aimed to identify factors associated with higher risk for CUD among PWH reporting recent cannabis use on a digital screening tool in primary care. Our study found that 35.8% of the sample were at higher risk for CUD. In the multivariable model, Black race, anxiety, and higher risk tobacco use were significantly associated with higher risk for CUD, while unadjusted models demonstrated several factors, including age, race, comorbidities, anxiety, depression, and tobacco use, were independently associated with this outcome.

The finding that race/ethnicity was a significant predictor of CUD risk has important implications. Disparities in HIV care for Black individuals [30] and the increased risk for cannabis-related consequences among Black individuals in the general population [31] are previously documented. Our results similarly suggest that Black PWH might be particularly at risk for consequences related to cannabis use. This finding is also notable given that high risk tobacco use was also a significant indicator of higher risk CUD. Prior studies find that Black PWH are more likely to endorse tobacco use [32]. Future work might examine comorbid risks associated with other substance use and cannabis use among Black PWH in particular. Ensuring that screening and intervention tools are culturally-adapted and accessible are critical for identifying and addressing higher risk cannabis use among Black PWH [33]. Relatedly, we found that higher risk tobacco use was associated with higher risk for CUD. Tobacco is highly prevalent among individuals with HIV [34] and co-use of cannabis and tobacco is common in general [4]. Interventions to address smoking among PWH may be particularly helpful for reducing use and mitigating harms related to both cannabis and tobacco use.

Mental health problems might also be impacted by cannabis use among PWH [3]. In our study, depression and anxiety were associated with greater odds of higher risk for CUD in unadjusted models, although only the recent anxiety symptom score was significant after adjustment for other factors. Possibly, PWH may use cannabis to specifically mitigate anxiety [35], relative to other mental health concerns, though additional research is needed to better understand the short-term and long-term consequences of cannabis and anxiety for PWH. Screening of mental health symptoms, particularly in a primary care setting that can be sensitive to changes over time, can provide opportunities for intervention and may prevent worsening concerns related to cannabis use among PWH.

Our study also found that age had an overall significant association with CUD risk. While a specific contrast did not emerge as significant, nevertheless, age is an important factor among PWH who use cannabis. While cannabis use is prevalent among young adults generally, recent trends show more older adults are initiating cannabis use, particularly to address aging-related health concerns such as pain [29]. Though older PWH may benefit from anti-inflammatory effects of cannabis use [36], further work is needed to address the implications of cannabis on HIV status and age, given the clinical implications of poorer treatment adherence among younger PWH broadly [37]. Although age only was significant in the univariate model, the population of PWH is rapidly aging, and providers need to consider cannabis use among patients across the lifespan [38, 39]. Further research is also needed to explore how age may interact with other relevant CUD risk factors, and if motivations and effects of cannabis use may change among PWH as they age, and in relation to other demographic factors.

Notably, we did not find associations between HIV indicators (duration of HIV diagnosis, HIV RNA levels, CD4 counts) and CUD risk. One study found that all measures of cannabis use (ever using during study period, any use, past week frequency) were associated with having detectable HIV RNA, though CD4 counts were comparable across cannabis use frequencies [6]. Participants in our study had HIV virologic control, were insured patients recruited from primary care clinics, and may have had better treatment adherence and management of their HIV symptoms compared with those in other settings, which might account for these null findings. Nevertheless, future work should continue to examine the relationship of HIV control to higher risk cannabis use.

### Limitations

Data were collected from PWH enrolled through primary care sites at KPNC. Findings may not generalize to other populations, including those receiving treatment in non-primary care settings, are uninsured, or residing in states with restrictive cannabis use policies. More than half of participants were White and the majority were male, and prior work has found that men are more likely to endorse riskier cannabis use [40] While this does underscore the importance of assessing CUD and cannabis use among male PWH, future analyses with samples that include greater numbers of women are crucial for further exploring how associations of higher risk cannabis use may relate to sociodemographic factors, such as among women and PWH with different racial/ethnic identities. Nevertheless, the sample was recruited from a health system that is representative of the insured population of Northern California.

While the TAPS is validated in primary care settings, as a brief screening instrument, items are limited in assessing different aspects of cannabis use. Sensitivity and specificity of the TAPS in identifying CUD risk may potentially differ among PWH and those with other chronic conditions (who may use cannabis medically) compared to the general population. Motivations for use, including medical use and coping are important to consider for PWH who may be using frequently for therapeutic benefit. Providers should be mindful of these patterns common to PWH when interpreting cannabis risk scores on the TAPS. Importantly, the TAPS is a brief assessment tool for identifying potential CUD, and further clinical assessment would be needed for a CUD diagnosis. Additionally, further work is needed to psychometrically validate this tool for PWH populations. Finally, the present analyses were limited to cross-sectional associations between HIV factors and cannabis use, and comments about causality cannot be made. Further research is needed to examine potential effects of HIV-related factors on changes in cannabis use over time, and could include additional variables, such as social support and other treatment factors to explore associations between HIV status and cannabis-related outcomes.

## Conclusion

This study aimed to identify CUD risk factors among PWH identified through systematic digital screening in primary care. PWH who were Black, and had elevated anxiety and tobacco use had increased odds of being at higher risk for CUD. Efforts should be made to continue monitoring mental health and substance use behaviors and address race-based disparities in cannabis use impacts among PWH.

## Data Availability

Data is unavailable for sharing.
